# PLAIN LANGUAGE SUMMARY

**DOI:** 10.2903/j.efsa.2023.p211001

**Published:** 2023-10-11

## Abstract

This publication is linked to the following EFSA Journal article: https://efsa.onlinelibrary.wiley.com/doi/10.2903/j.efsa.2023.8271

efsa JOURNAL

**10 October 2023**

## Vaccination of poultry against highly pathogenic avian influenza – Available vaccines and vaccination strategies

### Background to the scientific opinion

- The European Union (EU) has experienced an epidemic of highly pathogenic avian influenza (HPAI) in wild and domestic birds in recent years.
- Areas with dense populations of poultry farms are at particular risk of HPAI transmission.
- Vaccination can be considered in addition to already implemented measures (e.g. surveillance, biosecurity and early detections) to prevent and control the spread of HPAI.

### What was EFSA asked to do?

- The European Commission requested an overview of the available HPAI vaccines, their efficacy against currently circulating viruses and an assessment of various vaccination schemes to inform decisions on possible vaccination strategies.
- EFSA was also mandated to assess the surveillance and risk mitigation measures in vaccinated areas and farms. These topics will be addressed in a separate scientific opinion, with expected finalisation in March 2024.

### How did EFSA carry out this work?

- EFSA issued a scientific opinion on this topic in 2007: https://efsa.onlinelibrary.wiley.com/doi/abs/10.2903/j.efsa.2007.489
- In the current opinion, information about available vaccines and their characteristics was collected via a literature search, and in a survey that was launched by the European Medicines Agency (EMA) and disseminated by EFSA and the World Organisation for Animal Health (WOAH). Data were also collected through consultation of the EU Reference Laboratory (EURL) and the National Reference Laboratory (NRL) network, and directly from pharmaceutical companies' websites.
- The effectiveness of vaccination strategies was simulated using mathematical modelling.
- The data used were:
  - Published literature (there were no restrictions on publication language or study location).
  - Survey data.
  - Data on poultry population in France, Italy and the Netherlands.
  - Data on HPAI outbreaks in France, Italy and the Netherlands.
  - Data on pre-emptive culling in France, Italy and the Netherlands.

### What were the limitations/uncertainties?

- Data on protection efficacy and duration of protection of available vaccines against HPAI are non-harmonised and few; therefore, the detailed description and comparison of these vaccines is not possible.
- Few vaccines are tested on poultry species other than chickens.
- Field studies on the effectiveness of vaccination to stop virus transmission are scarce.

### What were the outcomes and their implications?

Outcomes

- Updated information was provided on types and characteristics of available vaccines against HPAI.
- There is only one authorised vaccine against HPAI in chickens in the EU.
- Insights were provided on different vaccination strategies for controlling HPAI in poultry, i.e. emergency protective vaccination in areas around a HPAI outbreak and preventive vaccination in areas and farms where the infection is not present yet.
- Recommendations were provided for future scientific studies on HPAI vaccines.

Implications

- EFSA's scientific advice on HPAI vaccines and vaccination strategies will inform EU Member State policy makers and risk managers about potential prevention and control strategies for HPAI, thus enabling informed decisions on HPAI prevention and outbreak control.
- The advice from EFSA may guide future development and use of vaccines against HPAI.

### What are the key recommendations?

Recommendations for policy makers and risk managers:

- Preventive vaccination is the optimal vaccination strategy to minimise the number of outbreaks and duration of epidemic and should be conducted in the most susceptible and infectious poultry species in high-risk transmission areas. Multiple administration (i.e. booster vaccinations) can be used to enhance protection.
- In case of an outbreak, emergency protective vaccination is recommended in a 3-km radius of the outbreak in high-risk transmission areas.
- Vaccine efficacy should be monitored for all vaccination strategies.
- Vaccination should complement and not replace other preventive and control measures, such as infection monitoring in birds, early detection and biosecurity, and is recommended as part of an integrated disease control approach.

Recommendations for the research community:

- The scientific opinion includes recommendations for future scientific studies on HPAI vaccines in terms of the types of vaccines to develop and for which bird species, the aspects of the vaccines that should be researched and the type of studies that should be conducted.

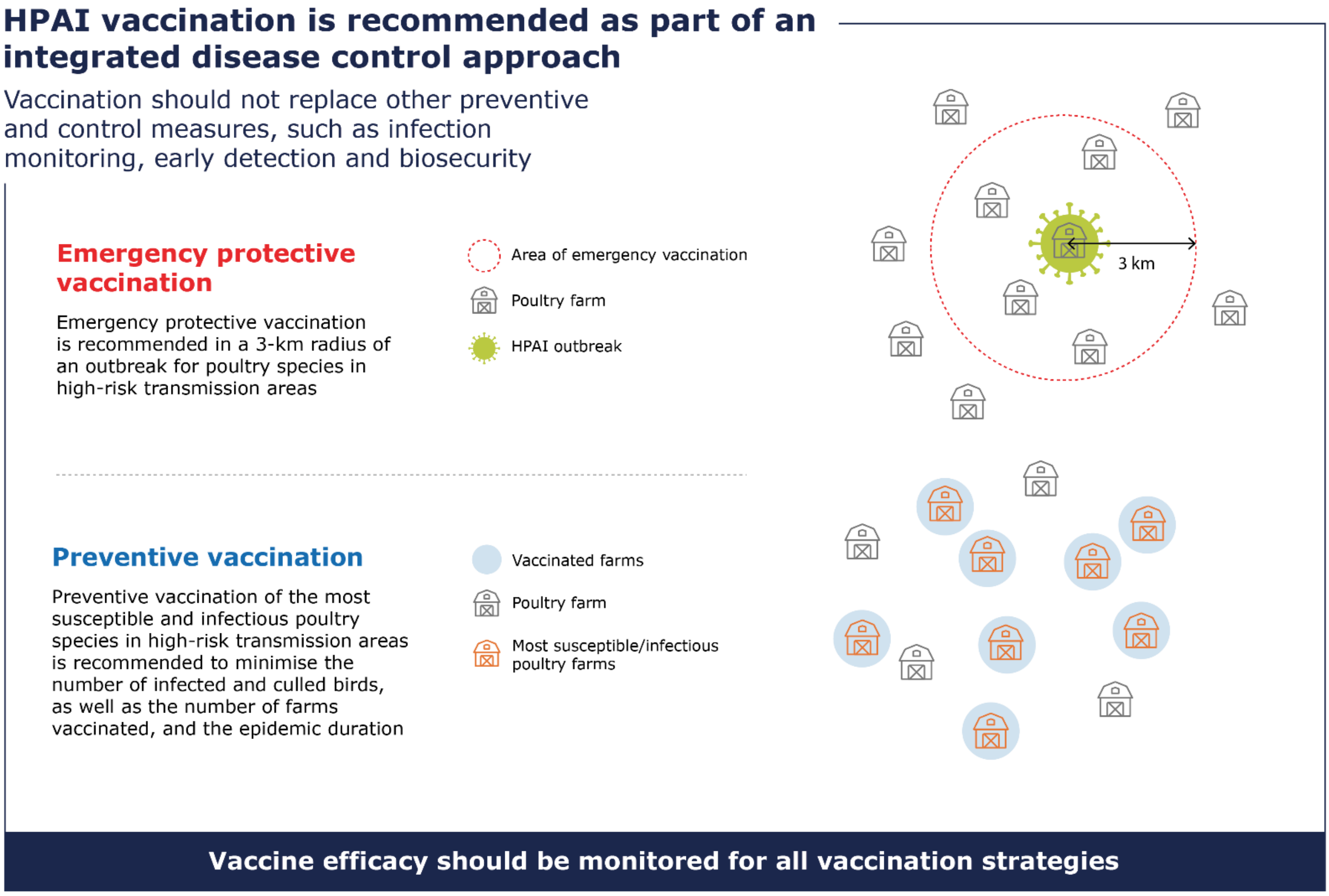

**Figure: Recommended strategies for HPAI emergency protective vaccination and preventive vaccination**

### Glossary

Available vaccines: inactivated vaccines or vaccines based on principles other than live attenuated avian influenza virus that have marketing authorisation; vaccines used inside or outside the EU; and vaccines in late-stage development for which performance data (laboratory or field) are available.

**Disclaimer**

- This plain language summary (PLS) is a simplified communication of EFSA's *Vaccination of poultry against highly pathogenic avian influenza – Part 1. Available vaccines and vaccination strategies*. The full EFSA opinion can be found here.
- The purpose of the PLS is to enhance transparency and inform interested parties on EFSA's work on the topic using simplified language to present a summary of the main findings.
-
